# Oxidative Stress during Ovarian Torsion in Pediatric and Adolescent Patients: Changing The Perspective of The Disease

**DOI:** 10.22074/ijfs.2015.4598

**Published:** 2015-12-23

**Authors:** Antonio Simone Laganà, Vincenza Sofo, Francesca Maria Salmeri, Vittorio Italo Palmara, Onofrio Triolo, Milan Milosav Terzić, Tito Silvio Patrelli, Adolf Lukanovic, Eda Vrtcnik Bokal, Giuseppe Santoro

**Affiliations:** 1 Unit of Gynecology and Obstetrics, Department of Human Pathology in Adulthood and Childhood, G. Barresi, University of Messina, Messina, Italy; 2 Department of Biomedical Sciences, Dentistry and Morphological and Functional Imaging, University of Messina, Messina, Italy; 3 University of Belgrade, School of Medicine, Belgrade, Serbia; 4 Clinic for Gynecology and Obstetrics, Clinical Center of Serbia, Belgrade, Serbia; 5 University of Parma, Parma, Italy; 6 Vicenza General Hospital, Vicenza, Italy; 7 Department of Gynecology and Obstetrics, University Clinical Center, Ljubljana, Slovenia; 8 Department of Biomedical Sciences and Morpho-Functional Images, University of Messina, Messina, Italy

**Keywords:** Paediatrics, Adolescents, Ischaemia/Reperfusion, Oxidative Stress, Antioxidants

## Abstract

Among the different causes of gynecological acute pelvic pain, ovarian torsion represents a surgical emergency. It is a rare case in the pediatric/adolescent aged group that must be included in the differential diagnosis of any girl with abdominal pain or pelvic/abdominal mass. Current recommendations suggest that laparoscopic detorsion should be performed in order to preserve the integrity of the ovaries and fertility, although oophoropexy may be considered in case of severe necrosis. Nevertheless, maintaining the circulation of the ovary after detorsion deteriorates the tissue injury and leads to a pathologic process called ischaemia/reperfusion (I/R) injury, which is characterized by oxidative stress. During the detorsion process, an excess amount of molecular oxygen is supplied to the tissues, and reactive species of oxygen (ROS) such as superoxide radical (O_2_^-^), hydrogen peroxide (H_2_O_2_), hydroxyl radical (OH•), as well as reactive nitrogen species (RNS) are produced in excess. ROS, RNS and their toxic products cause DNA damage and lipid peroxidation in the cellular and mitochondrial membranes, leading to cell death. In spite of attention on this topic, currently there is no shared and clear evidence about the use of anti-inflammatory and antioxidant agents to prevent I/R damage after laparoscopic ovarian detorsion. Considering this element, future research should aim to develop shared protocols for the clinical use (route of application, dosage and time of application) of antioxidants after laparoscopic management of this condition.

## Introduction

### Ovarian torsion in pediatric and adolescent patients: what to do?

Among the different causes of gynecological acute pelvic pain, ovarian torsion (1) represents a surgical emergency. It occurs in 2.7% of all pediatric/adolescent population who presents with acute abdominal pain (2,3). In this regard, torsion of the fallopian tube without torsion of the ipsilateral ovary, as known as Isolated Tubal Torsion (ITT), is an extremely rare event (4,5) which may be caused by extremely large Morgagni hydatid (6). Furthermore, ovarian torsion must be considered in the differential diagnosis of any girl with abdominal pain or pelvic/ abdominal mass (7,8). Adnexal masses are uncommon events in the pediatric/adolescent population. The estimated incidence is approximately 2.6 per 100,000 girls younger than 18 years of age (9) and 10% of pediatric ovarian masses are found to be malignant (10,12). 

According to Millar et al. (13) ovarian cysts are found in 2-5% of prepubertal females undergoing ultrasound scan , although they are mostly small (<1 cm) and insignificant. Considering only the neoplastic disease, it has been estimated that approximately 67% of ovarian cancers in childhood and adolescence are originated from germ cells (14), whereas the classic epithelial ovarian cancer occurs very rarely in pre-menarche (15). In the post-pubertal age, functional ovarian cysts could occur under several conditions: anovulation, excessive stimulation by follicle-stimulating hormone (FSH) or lack of normal luteinizing hormone (LH) peak, estrogens excess without corresponding progesterone influence (13,16,17). 

Clinical presentation may comprise spotting of moderate vaginal bleeding and acute/chronic pelvic pain (18). In particular, the failure of luteolysis 14 days after the ovulation could cause persistence of theca-lutein cyst, and this condition could also occur in pregnancy for the high level of human chorionic gonadotropin (hCG) which cause hypertrophy of theca cells (19). About 3% of theca-lutein cysts are complicated by torsion or hemorrhage and 30% of these can cause hyperandrogenism (17). Furthermore, the other kind of ovarian cysts may be complicated with infarction and necrosis (20). Adnexal torsion in pediatric/adolescent population is most commonly (approximately 97%) caused by a benign ovarian cyst or teratoma (21). The size of an ovarian cyst has not been shown to correlate with an increased risk of ovarian torsion (22); moreover, ovarian torsions are more likely to result in the right side due to the protective effect of the sigmoid colon (23). 

On ultrasound scan, an enlarged ovary and increased volume ratio in comparison to the contralateral ovary is indicative of an ovarian torsion (24). In this regard, ultrasound scan remains the most useful investigation (25,28), although in pediatric/adolescent population blood flow on Doppler examination does not exclude ovarian torsion (18,23,29) and sometimes computed tomography (CT) scan is needed (20). Current recommendations of treatment strongly support ovary conservation since macroscopic appearance of the ovary is not a reliable indicator of the degree of necrosis and potentiality for ovarian recovery (8). For children/adolescent with ovarian torsion, laparoscopic detorsion should be performed in order to preserve the integrity of the ovaries and fertility, although oophoropexy may be considered in case of severe necrosis (30,31). 

Nevertheless, in case of laparoscopic detorsion, it must be taken into account that the restoring of ovarian blood flow may be only partial and slow, and in addition to that, the ovarian damage could progressively increase for oxidative stress after reperfusion (32). Considering all these elements, early diagnosis and surgical intervention are essential, especially in adolescents, in order to preserve the anatomy and function of ovaries (1,33). 

### Reactive oxygen species and cellular microenvironment: master and minion

Reactive oxygen species (ROS), derived from molecular oxygen, may present as free radicals or other forms; they have electronically unstable and ionized atomic structure, which interacts with biological macromolecules by capturing electrons and interfering with their biological functionality (34-37). There are several enzymatic activities involved in the generation of ROS: the reduction of dioxygen (O_2_) in the mitochondria can lead to various intermediate forms of ROS; within peroxisomes, the reduction of amino acid oxidase triggers the oxidative deamination in the α-keto acids and copper and iron ions generate ROS that may be released in to the blood flow by transferrin, albumin, and ceruloplasmin.

Finally, as well as intracellular nicotinamide adenine dinucleotide phosphate [NAD(P)H]-oxidase, cyclooxygenase, nitric oxide synthase (NOS) and xanthine oxidase produce ROS (38-40). Reactive nitrogen species (RNS) include nitric oxide (NO), nitrogen dioxide (NO_2_) and non-reactive species such as peroxynitrite (ONOO−), and nitrosamines (41). In mammals, RNS are mainly derived from NO, which is formed by NOS from O_2_ and L-arginine using NADPH as an electron donor (42), and from its reaction with the superoxide anion, which forms peroxynitrite (43). Peroxynitrite could trigger lipid peroxidation and nitrosation of several tyrosine molecules, which play a key role in enzyme function and signal transduction pathways (41). Moreover, accumulating evidence suggests that the activity of endothelial NOS (eNOS) is increased in response to the LH surge and hCG (44). Moreover, animal models showed that eNOS expression is enhanced during chronic hypoxic conditions (45, 46).

Conversely, it has been demonstrated that hypertension in humans may occur due to suboptimal vascular endothelial production of NO (47). The physiological function of ROS has been widely studied in monocyte-macrophages, granulocytes, natural killer (NK) cells and neutrophils.Within these cells, there is a clear evidence that the NAD(P)H oxidase, the myeloperoxidase and the NOS produce anion superoxide, hypochlorous acid and nitrogen monoxide, triggering the cytotoxic responses of the immune system (38,40). Furthermore, oxidizing agents play a key role in the phosphorylation and activation of protein kinases, and in the phospholipase signaling pathways mediated by Ca^++^. In this regard, oxidative stress activates phosphorylated proteins. Considering the point that the activity of these protein kinases are strictly involved in the cell cycle regulation, it has been showed that the inhibition of these transduction cascades by ROS scavenger and antioxidant agents, such as superoxide dismutase (SOD), can inhibit the cell cycle progression (48). 

### Markers of oxidative stress: causes and effects

Since ROS are potentially harmful, the human body has evolved highly complex antioxidant defense systems, both enzymatic and non-enzymatic, which synergistically function in combination. Some antioxidants are produced directly in the intracellular microenvironment (enzymatic antioxidants), such as glutathione oxidase and peroxidase, ubiquinone (CoQ10), α-lipoic acid (ALA), SOD and superoxide catalase (49), while the non-enzymatic antioxidants consist of dietary supplements and synthetic antioxidants such as vitamin C, glutathione, taurine, hypotaurine, vitamin E, Zn, selenium (Se), betacarotene, and carotene (34,50,51). 

Mechanism of antioxidants’ action depends on their concentration, which is variably presented in fluids and tissues. Multiple redox reactions occur within cell metabolism which can lead to oxidative stress if unbalanced in homeostatic mechanisms. Although cells have several intrinsic antioxidant mechanisms, when ROS are present in large amount, the ability to rebalance homeostasis is exceeded and as a result cellular damage may occur (34,37). An excess of ROS may cause a cascade of events such as the release of Ca ^++^ which results in mitochondrial permeability and provokes mitochondrial membrane instability and consequent cessation of adenosine triphosphate (ATP) production; the lipid peroxidation, which increase the peroxyl radicals, damage of amino acids which leads to the formation of carbonyl groups. Oxidation of the mitochondrial DNA (mtDNA) without protection of histones, does not own any repair mechanisms (43). As result of this complex mechanisms , oxidative stress finally causes DNA damage and/ or apoptosis of the cell (52). The total antioxidant status (TAS) was employed to assess the general antioxidative status (53). 

Accordingly, total oxidant status (TOS) is obtained to ascertain the overall oxidation status. Represented as the ratio of TOS to TAS, the oxidative stress index (OSI) considered a precise index of oxidative stress (54). As meticulously reviewed by Agarwal et al. (49) oxidative stress seems to play a key role in the physiologic processes of menstrual cycle and ovulation, embryo implant, placental framework development, menopause. Conversely, accumulating evidence suggest that a breakdown in red-ox homeostasis occurs during several reproductive disease such as endometriosis, polycystic ovary syndrome, unexplained infertility, spontaneous abortion, recurrent pregnancy loss, preeclampsia, intrauterine growth restriction and preterm labor. In particular, this review will try to shed light on the redo-ox process during the adnexal torsion in pediatric and adolescent patients. 

### Oxidative stress during ovarian torsion in pediatric and adolescent patients

The ovaries are organs of constantly predictable change during the reproductive cycle. Their arterial supply comes from two different vases that anastomose in the mesovarium: the ovarian artery, branch of the abdominal aorta, and the ovarian branch of the uterine artery, that ascends the uterus tortuously to reach the mesovarium. Although ovarian torsion may occur most often in the first 3 decades in normal ovaries, it is more frequent in association with pre-existing tubal/ovarian pathologies (55) for this reason it is a condition which rarely occurs in pediatric/adolescent patients. There might be more than one pathophysiological mechanism for ovarian torsion in these patients: the most important of which are growth of ovarian volume and excessive mobility of the tube and mesosalpinx with long ovarian ligaments (56). 

Furthermore, several Authors (57,58) suggest an average age of 10 years for occurring this condition. The time of the tissue’s exposure to ischemia is critical. According to Macdougall (59), it is possible to have restitutio ad integrum of the adnexa following reperfusion within 18-24 hours after blood supply interruption. Moreover, ovarian torsion is not an isolated disease: Also unilateral torsion and ovariectomy affect the ovulation in contralateral ovary (60,62). Indeed, Akgur et al. (62) reported histological and ultrastructural changes in contralateral ovaries after ipsilateral ovarian ischaemia. This could be due, at least in part, to the stimulation of the sympathetic system by unilateral ovarian ischaemia, which may cause reduction of regional blood flow similar to testicular torsion. The tissue damage in the twisted ovary depends on one hand to direct damage caused by ischaemia during torsion and, on the other hand, to a secondary effect due to reperfusion during detorsion as a common mechanism to other organs such as kidney and brain (63,64). Considering that because of release of thrombi from a thrombotic pelvic vein after detorsion of the twisted adnexa, thromboembolic phenomenon has never been demonstrated (65,67). 

Nowadays to date laparoscopic ovarian detorsion is preferred with respect to ovariectomy especially in pediatric/adolescent patients (68,70). In particular, according to Galinier et al. (7) conservative approach of black-bluish ovaries after detorsion is safe and effective. Moreover, these authors also indicate that the size of abdominopelvic mass could be used as a good discriminating indicator to classify patients in the correct surgical procedure group before surgery. If it is lower than 75 mm, laparoscopy is preferred, whereas if it is equal to or greater than 75 mm, laparotomy is preferred. There is neither consensus about oophoropexy after untwisting, nor the preventive pexy on the opposite side (71,72), even if it is advisable to perform this technique when the mesosalpinx is clearly loose (57). 

Celik et al. (73) showed that after conservative treatment of 14 ovarian torsion, 13 of 14 ovaries that had been conserved. This procedure showed echographic imaging of follicullogenesis without ovarian atrophy. Others (74) underlined the lack of correlation between duration of symptoms and ovarian infarction. Finally, few pediatric observations of asynchronous bilateral torsions treated by ovarian removal and simple untwisting of the other have been published, although it could be considered a viable option in this condition (74,76). Nevertheless, maintaining the circulation of the ovary after detorsion makes the tissue injury worse and leads to a pathologic process called ischaemia/reperfusion (I/R) injury, characterized by oxidative stress (77). During the detorsion process, an excess amount of molecular oxygen is supplied to the tissues, and ROS such as superoxide radical (O_2_^-^), hydrogen peroxide (H_2_O_2_), hydroxyl radical (OH•), and NO are produced in excess. ROS, RNS and their toxic products cause DNA damage and lipid peroxidation in the cellular and mitochondrial membranes (77,79). 

As reported by Ozat et al. (80), during I/R injury a massive influx of Ca ^++^ into the cell occurs, caused by the action of sarcolemma and release of this cation from intracellular binding/sequestration sites 81,82). Moreover, the excess of cytosolic Ca ^++^ triggers several enzymatic pathways which provokes an inflammatory status and accumulation of free arachidonic acid and leads to the depletion of Ca ^++^from the endoplasmic reticulum lumen (64,82,84). Following these events, the intracellular excess Ca ^++^causes the release of cytochrome c from mitochondria and activation of caspase-dependent and caspase-independent cell death (8,85,86). During I/R injury, the most important mediator which is released in this process, and contribute as biomarkers of oxidative stress, is malondialdehyde (MDA) (87,88). Till now, a number of antiinflammatory and antioxidant agents have been used to prevent I/R injury, most of them in animal models (89,90): SOD (91), curcumin (2,92,93), iloprost (a prostacyclin analogue) (80), melatonin (94), aprotinin (95), recombinant erythropoietin (rhEPO) (67,96,97), calcium channel blockers (98,99) as 2-Aminoethoxydiphenyl borate (2-APB) (86,100,102), growth hormone (GH) (103), ozone (O_3_) therapy (104), dimethylsulfoxide (DMSO) (105), alpha-lipoic acid (LA, 1, 2-dithiolane-3-pentanoic acid) (106), genistein (107), marrubium cordatum (108) and amlodipine (109). Despite of the increasing attention to this topic, currently there is no shared and clear evidence about the use of anti-inflammatory and antioxidant agents to prevent I/R damage after laparoscopic ovarian detorsion. According to our literature review, future researches should focus on the correlation between I/R injury and consequent cellular/molecular events, especially by taking into account the role of the alterations in cellular immunity (110) and the apoptosis pathways (111,112). 

## Conclusion

Current evidence suggests to performing laparoscopic ovarian detorsion, with respect to oophorectomy, in case of ovarian torsion in pediatric and adolescent patients. Although this procedure is acceptable, we must keep in mind that I/R injury can extend and make worse the ischemic and necrotic damage. Considering this element, future research should aim to develop shared protocols for the clinical use (route of application, dosage and time of application) of antioxidants after laparoscopic ovarian detorsion. 

## References

[1] Al-Shukri M, Mathew M, Al-Ghafri W, Al-Kalbani M, Al- Kharusi L, Gowri V (2014). A clinicopathological study of women with adnexal masses presenting with acute symptoms. Ann Med Health Sci Res.

[2] Sak ME, Soydinc HE, Sak S, Evsen MS, Alabalik U, Akdemir F (2013). The protective effect of curcumin on ischemia- reperfusion injury in rat ovary. Int J Surg.

[3] Hibbard LT (1985). Adnexal torsion. Am J Obstet Gynecol.

[4] Huchon C, Fauconnier A (2010). Adnexal torsion: a literature review. Eur J Obstet Gynecol Reprod Biol.

[5] Erdemoğlu M, Kuyumcuoglu U, Guzel AI (2011). Clinical experience of adnexal torsion: evaluation of 143 cases. J Exp Ther Oncol.

[6] Terzic M, Arsenovic N, Maricic S, Babovic I, Pilic I, Bila J (2011). Fallopian tube torsion caused by extremely large Morgagni hydatid: a very rare cause of acute abdomen in a virgin adolescent. Cent Eur J Med.

[7] Galinier P, Carfagna L, Delsol M, Ballouhey Q, Lemasson F, Le Mandat A (2009). Ovarian torsion.Management and ovarian prognosis: a report of 45 cases. J Pediatr Surg.

[8] Cass DL (2005). Ovarian torsion. Semin Pediatr Surg.

[9] Skinner MA, Schlatter MG, Heifetz SA, Grosfeld JL (1993). Ovarian neoplasms in children. Arch Surg.

[10] Oltmann SC, Garcia N, Barber R, Huang R, Hicks B, Fischer A (2010). Can we preoperatively risk stratify ovarian masses for malignancy?. J Pediatr Surg.

[11] Cass DL, Hawkins E, Brandt ML, Chintagumpala M, Bloss RS, Milewicz AL (2001). Surgery for ovarian masses in infants, children, and adolescents: 102 consecutive patients treated in a 15-year period. J Pediatr Surg.

[12] Templeman C, Fallat ME, Blinchevsky A, Hertweck SP (2000). Noninflammatory ovarian masses in girls and young women. Obstet Gynecol.

[13] Millar DM, Blake JM, Stringer DA, Hara H, Babiak C (1993). Prepubertal ovarian cyst formation: 5 years’ experience. Obstet Gynecol.

[14] You W, Dainty LA, Rose GS, Krivak T, McHale MT, Olsen CH (2005). Gynecologic malignancies in women aged less than 25 years. Obstet Gynecol.

[15] Brown MF, Hebra A, McGeehin K, Ross AJ 3rd (1993). Ovarian masses in children a review of 91 cases of malignant and benign masses. J Pediatr Surg.

[16] Katz VL, Lentz GM, Lobo RA, Gershenson DM (2007). Comprehensive gynecology.

[17] Stany MP, Hamilton CA (2008). Benign disorders of the ovary. Obstet Gynecol Clin North Am.

[18] Choudry A, Bangash N, Malik A, Choudry H (2008). Adolescent ovarian tumors: a clinicopathlogical review of 15 cases. J Ayub Med Coll Abbottabad.

[19] Stephen GL, Willet MJ (2009). Theca-lutein cysts complicating recovery after termination of a triploid pregnancy. J Obstet Gynaecol.

[20] Glanc P, Salem S, Farine D (2008). Adnexal masses in the pregnant patient: a diagnostic and management challenge. Ultrasound Q.

[21] Kokoska ER, Keller MS, Weber TR (2000). Acute ovarian torsion in children. Am J Surg.

[22] Warner BW, Kuhn JC, Barr LL (1992). Conservative management of large ovarian cysts in children: the value of serial pelvic ultrasonography. Surgery.

[23] Surratt JT, Siegel MJ (1991). Imaging of pediatric ovarian masses. Radiographics.

[24] Servaes S, Zurakowski D, Laufer MR, Feins N, Chow JS (2007). Sonographic findings of ovarian torsion in children. Pediatr Radiol.

[25] Terzic M, Dotlic J, Brndusic N, Arsenovic N, Likic I, Ladjevic N (2013). Histopathological diagnoses of adnexal masses: which parameters are relevant in preoperative assessment?. Ginekol Pol.

[26] Terzic M, Dotlic J, Likic I, Ladjevic N, Brndusic N, Mihailovic T (2013). Predictive factors of malignancy in patients with adnexal masses. Eur J Gynaecol Oncol.

[27] Terzic MM, Dotlic J, Likic I, Ladjevic N, Brndusic N, Arsenovic N (2013). Current diagnostic approach to patients with adnexal masses: which tools are relevant in routine praxis?. Chin J Cancer Res.

[28] Dotlic J, Terzić M, Likic I, Atanackovic J, Ladjevic N (2011). Evaluation of adnexal masses: correlation between clinical, ultrasound and histopathological findings. Vojnosanit Pregl.

[29] Deligeoroglou E, Eleftheriades M, Shiadoes V, Botsis D, Hasiakos D, Kontoravdis A (2004). Ovarian masses during adolescence: clinical, ultrasonographic and pathologic findings, serum tumor markers and endocrinological profile. Gynecol Endocrinol.

[30] Parker WH, Berek JS (1993). Management of the adnexal mass by operative laparoscopy. Clin Obstet Gynecol.

[31] Eckler K, Laufer MR, Perlman SE (2000). Conservative management of bilateral asynchronous adnexal torsion with necrosis in a prepubescent girl. J Pediatr Surg.

[32] Webb EM, Green GE, Scoutt LM (2004). Adnexal mass with pelvic pain. Radiol Clin North Am.

[33] Zolton JR, Maseelall PB (2013). Evaluation of ovarian cysts in adolescents. Open J Obstet Gynecol.

[34] Meister A (1988). Glutathione metabolism and its selective modification. J Biol Chem.

[35] Simmons SO, Fan CY, Ramabhadran R (2009). Cellular stress response pathway system as a sentinel ensemble in toxicological screening. Toxicol Sci.

[36] Kultz D (2005). Molecular and evolutionary basis of the cellular stress response. Annu Rev Physiol.

[37] Thannickal VJ, Fanburg BL (2000). Reactive oxygen species in cell signaling. Am J Physiol Lung Cell Mol Physiol.

[38] Paravicini TM, Touyz RM (2008). NADPH oxidases, reactive oxygen species, and hypertension: clinical implications and therapeutic possibilities. Diabetes Care.

[39] Jones DP (2008). Radical-free biology of oxidative stress. Am J Physiol Cell Physiol.

[40] Rabelo LA, Souza VN, Fonseca LJ, Sampaio WO (2010). Redox unbalance: NADPH oxidase as therapeutic target in blood pressure control. Arq Bras Cardiol.

[41] Rosselli M, Keller PJ, Dubey RK (1998). Role of nitric oxide in the biology, physiology and pathophysiology of reproduction. Hum Reprod Update.

[42] Ignarro LJ (2002). Nitric oxide as a unique signaling molecule in the vascular system: a historical overview. J Physiol Pharmacol.

[43] Burton GJ, Jauniaux E (2011). Oxidative stress. Best Pract Res Clin Obstet Gynaecol.

[44] Fujii J, Iuchi Y, Okada F (2005). Fundamental roles of reactive oxygen species and protective mechanisms in the female reproductive system. Reprod Biol Endocrinol.

[45] Ducsay CA, Myers DA (2011). eNOS activation and NO function: differential control of steroidogenesis by nitric oxide and its adaptation with hypoxia. J Endocrinol.

[46] Xiao D, Bird IM, Magness RR, Longo LD, Zhang L (2001). Upregulation of eNOS in pregnant ovine uterine arteries by chronic hypoxia. Am J Physiol Heart Circ Physiol.

[47] Versari D, Daghini E, Virdis A, Ghiadoni L, Taddei S (2009). Endothelium-dependent contractions and endothelial dysfunction in human hypertension. Br J Pharmacol.

[48] Preston TJ, Muller WJ, Singh G (2001). Scavenging of extracellular H2O2 by catalase inhibits the proliferation of HER-2/ Neu-transformed rat-1 fibroblasts through the induction of a stress response. J Biol Chem.

[49] Agarwal A, Aponte-Mellado A, Premkumar BJ, Shaman A, Gupta S (2012). The effects of oxidative stress on female reproduction: a review. Reprod Biol Endocrinol.

[50] Diplock AT, Charleux JL, Crozier-Willi G, Kok FJ, Rice- Evans C, Roberfroid M (1998). Functional food science and defence against reactive oxidative species. Br J Nutr.

[51] Agarwal A, Gupta S, Sharma RK (2005). Role of oxidative stress in female reproduction. Reprod Biol Endocrinol.

[52] Lee JY, Baw CK, Gupta S, Aziz N, Agarwal A (2010). Role of oxidative stress in polycystic ovary syndrome. Curr Womens Health Rev.

[53] Erel O (2004). A novel automated method to measure total antioxidant response against potent free radical reactions. Clin Biochem.

[54] Erel O (2005). A new automated colorimetric method for measuring total oxidant status. Clin Biochem.

[55] McWilliams GD, Hill MJ, Dietrich CS 3rd (2008). Gynecologic emergencies. Surg Clin North Am.

[56] Rousseau V, Massicot R, Darwish AA, Sauvat F, Emond S, Thibaud E (2008). Emergency management and conservative surgery of ovarian torsion in children: a report of 40 cases. J Pediatr Adolesc Gynecol.

[57] Beaunoyer M, Chapdelaine J, Bouchard S, Ouimet A (2004). Asynchronous bilateral ovarian torsion. J Pediatr Surg.

[58] Aziz D, Davis V, Allen L, Langer JC (2004). Ovarian torsion in children: is oophorectomy necessary?. J Pediatr Surg.

[59] Macdougall IC (2008). Novel erythropoiesis-stimulating agents: a new era in anemia management. Clin J Am Soc Nephrol.

[60] Coleman DA, Fleming MW, Dailey RA (1984). Factors affecting ovarian compensation after unilateral ovariectomy in gilts. J Anim Sci.

[61] Cakmak M, Kaya M, Barlas M, Dindar H, Gokçora H, Konkan R (1993). Histologic and ultrastructural changes in the contralateral ovary in unilateral ovarian torsion: an experimental study in rabbits. Tokai J Exp Clin Med.

[62] Akgur FM, Kilinç K, Tanyel FC, Buyukpamukçu N, Hiçsonmez A (1994). Ipsilateral and contralateral testicular biochemical acute changes after unilateral testicular torsion and detorsion. Urology.

[63] Ratych RE, Bulkley GB (1986). Free-radical-mediated postischemic reperfusion injury in the kidney. J Free Radic Biol Med.

[64] White BC, Sullivan JM, DeGracia DJ, O'Neil BJ, Neumar RW, Grossman LI (2000). Brain ischemia and reperfusion: molecular mechanisms of neuronal injury. J Neurol Sci.

[65] Oelsner G, Bider D, Goldenberg M, Admon D, Mashiach S (1993). Long-term follow-up of the twisted ischemic adnexa managed by detorsion. Fertil Steril.

[66] McGovern PG, Noah R, Koenigsberg R, Little AB (1999). Adnexal torsion and pulmonary embolism: case report and review of the literature. Obstet Gynecol Surv.

[67] Bakan V, Ciralik H, Tolun FI, Atli Y, Mil A, Ozturk S (2009). Protective effect of erythropoietin on torsion/detorsion injury in rat model. J Pediatr Surg.

[68] Way S (1946). Ovarian cystectomy of twisted cysts. Lancet.

[69] Shalev J, Goldenberg M, Oelsner G, Ben-Rafael Z, Bider D, Blankstein J (1989). Treatment of twisted ischemic adnexa by simple detorsion. N Engl J Med.

[70] Zweizig S, Perron J, Grubb D, Mishell DR Jr (1993). Conservative management of adnexal torsion. Am J Obstet Gynecol.

[71] Abeş M, Sarihan H (2004). Oophoropexy in children with ovarian torsion. Eur J Pediatr Surg.

[72] Thibaud E, Ramirez M, Brauner R, Flamant F, Zucker JM, Fekete C (1992). Preservation of ovarian function by ovarian transposition performed before pelvic irradiation during childhood. J Pediatr.

[73] Celik A, Ergun O, Aldemir H, Ozcan C, Ozok G, Erdener A (2005). Long-term results of conservative management of adnexal torsion in children. J Pediatr Surg.

[74] Anders JF, Powell EC (2005). Urgency of evaluation and outcome of acute ovarian torsion in pediatric patients. Arch Pediatr Adolesc Med.

[75] Ozcan C, Celik A, Ozok G, Erdener A, Balik E (2002). Adnexal torsion in children may have a catastrophic sequel: asynchronous bilateral torsion. J Pediatr Surg.

[76] Rody A, Jackisch C, Klockenbusch W, Heinig J, Coenen- Worch V, Schneider HP (2002). The conservative management of adnexal torsion--a case-report and review of the literature. Eur J Obstet Gynecol Reprod Biol.

[77] Li C, Jackson RM (2002). Reactive species mechanisms of cellular hypoxia-reoxygenation injury. Am J Physiol Cell Physiol.

[78] McCord JM (1985). Oxygen-derived free radicals in postischemic tissue injury. N Engl J Med.

[79] Grace PA (1994). Ischaemia-reperfusion injury. Br J Surg.

[80] Ozat M, Gungor T, Barun S, Demirogullari B, Sokmensuer LK, Gulbahar O (2009). The effects of iloprost, a prostacyclin analogue, in experimental ischaemia/reperfusion injury in rat ovaries. Exp Toxicol Pathol.

[81] Satoh H, Hayashi H, Katoh H, Terada H, Kobayashi A (1995). Na+/H+ and Na+/Ca2+ exchange in regulation of [Na+] i and [Ca2+]i during metabolic inhibition. Am J Physiol.

[82] Berna N, Arnould T, Remacle J, Michiels C (2001). Hypoxia-induced increase in intracellular calcium concentration in endothelial cells: role of the Na(+)-glucose cotransporter. J Cell Biochem.

[83] Du C, Koretsky AP, Izrailtyan I, Benveniste H (2005). Simultaneous detection of blood volume, oxygenation, and intracellular calcium changes during cerebral ischemia and reperfusion in vivo using diffuse reflectance and fluorescence. J Cereb Blood Flow Metab.

[84] Farber JL (1990). The role of calcium in lethal cell injury. Chem Res Toxicol.

[85] Vanlangenakker N, Vanden Berghe T, Krysko DV, Festjens N, Vandenabeele P (2008). Molecular mechanisms and pathophysiology of necrotic cell death. Curr Mol Med.

[86] Nicoud IB, Knox CD, Jones CM, Anderson CD, Pierce JM, Belous AE (2007). 2-APB protects against liver ischemiareperfusion injury by reducing cellular and mitochondrial calcium uptake. Am J Physiol Gastrointest Liver Physiol.

[87] Del Rio D, Stewart AJ, Pellegrini N (2005). A review of recent studies on malondialdehyde as toxic molecule and biological marker of oxidative stress. Nutr Metab Cardiovasc Dis.

[88] Carden DL, Granger DN (2000). Pathophysiology of ischaemiareperfusion injury. J Pathol.

[89] Kuroda S, Siesjo BK (1997). Reperfusion damage following focal ischemia: pathophysiology and therapeutic windows. Clin Neurosci.

[90] Land WG (2005). The role of postischemic reperfusion injury and other nonantigen-dependent inflammatory pathways in transplantation. Transplantation.

[91] Baker GL, Corry RJ, Autor AP (1985). Oxygen free radical induced damage in kidneys subjected to warm ischemia and reperfusion.Protective effect of superoxide dismutase. Ann Surg.

[92] Srivastava G, Mehta JL (2009). Currying the heart: curcumin and cardioprotection. J Cardiovasc Pharmacol Ther.

[93] Aggarwal BB, Harikumar KB (2009). Potential therapeutic effects of curcumin, the anti-inflammatory agent, against neurodegenerative, cardiovascular, pulmonary, metabolic, autoimmune and neoplastic diseases. Int J Biochem Cell Biol.

[94] Turkoz Y, Celik O, Hascalik S, Cigremis Y, Hascalik M, Mizrak B (2004). Melatonin reduces torsion-detorsion injury in rat ovary: biochemical and histopathologic evaluation. J Pineal Res.

[95] Sahin FK, Cosar E, Koken G, Toy H, Basarali K, Buyukbas S (2008). Protective effect of aprotinin on ischemia-reperfusion injury in rat ovary. J Obstet Gynaecol Res.

[96] Karaca M, Odabasoglu F, Kumtepe Y, Albayrak A, Cadirci E, Keles ON (2009). Protective effects of erythropoietin on ischemia/ reperfusion injury of rat ovary. Eur J Obstet Gynecol Reprod Biol.

[97] Sayyah-Melli M, Rashidi MR, Kaseb-Ganeh M, Rashtchizadeh N, Taghavi S, Ouladsahebmadarek E (2012). The effect of erythropoietin against oxidative damage associated with reperfusion following ovarian detorsion. Eur J Obstet Gynecol Reprod Biol.

[98] Sagsoz N, Kisa U, Apan A (2002). Ischaemia-reperfusion injury of rat ovary and the effects of vitamin C, mannitol and verapamil. Hum Reprod.

[99] Malis CD, Bonventre JV (1986). Mechanism of calcium potentiation of oxygen free radical injury to renal mitochondria.A model for post-ischemic and toxic mitochondrial damage. J Biol Chem.

[100] Taskin MI, Hismiogullari AA, Yay A, Adali E, Gungor AC, Korkmaz GO (2014). Effect of 2-aminoethoxydiphenyl borate on ischemia-reperfusion injury in a rat ovary model. Eur J Obstet Gynecol Reprod Biol.

[101] Gysembergh A, Lemaire S, Piot C, Sportouch C, Richard S, Kloner RA (1999). Pharmacological manipulation of Ins(1,4,5)P3 signaling mimics preconditioning in rabbit heart. Am J Physiol.

[102] Chinopoulos C, Starkov AA, Fiskum G (2003). Cyclosporin Ainsensitive permeability transition in brain mitochondria: inhibition by 2-aminoethoxydiphenyl borate. J Biol Chem.

[103] Yigiter M, Halici Z, Odabasoglu F, Keles ON, Atalay F, Unal B (2011). Growth hormone reduces tissue damage in rat ovaries subjected to torsion and detorsion: biochemical and histopathologic evaluation. Eur J Obstet Gynecol Reprod Biol.

[104] Aslan MK, Boybeyi O, Şenyucel MF, Ayva S, Kısa U, Aksoy N (2012). Protective effect of intraperitoneal ozone application in experimental ovarian ischemia/reperfusion injury. J Pediatr Surg.

[105] Ergun Y, Koc A, Dolapcioglu K, Akaydin Y, Dogruer G, Kontas T (2010). The protective effect of erythropoietin and dimethylsulfoxide on ischemia-reperfusion injury in rat ovary. Eur J Obstet Gynecol Reprod Biol.

[106] Cosar E, Sahin FK, Koken G, Toy H, Basarali K, Buyukbas S (2007). The protective effect of alpha-lipoic acid in experimental ovarian ischaemia-reperfusion injury. Aust N Z J Obstet Gynaecol.

[107] Yazici G, Erdem O, Cimen B, Arslan M, Tasdelen B, Cinel I (2007). Genistein attenuates postischemic ovarian injury in a rat adnexal torsion-detorsion model. Fertil Steril.

[108] Cigremis Y, Kart A, Karaman M, Erdag D (2010). Attenuation of ischemia-reperfusion injury with Marrubium cordatum treatment in ovarian torsion-detorsion model in rabbits. Fertil Steril.

[109] Halici Z, Karaca M, Keles ON, Borekci B, Odabasoglu F, Suleyman H (2008). Protective effects of amlodipine on ischemiareperfusion injury of rat ovary: biochemical and histopathologic evaluation. Fertil Steril.

[110] Laganà AS, Sturlese E, Retto G, Sofo V, Triolo O (2013). Interplay between misplaced müllerian-derived stem cells and peritoneal immune dysregulation in the pathogenesis of endometriosis. Obstet Gynecol Int.

[111] Sturlese E, Salmeri FM, Retto G, Pizzo A, De Dominici R, Ardita FV (2011). Dysregulation of the Fas/FasL system in mononuclear cells recovered from peritoneal fluid of women with endometriosis. J Reprod Immunol.

[112] Salmeri FM, Laganà AS, Sofo V, Triolo O, Sturlese E, Retto G (2015). Behavior of tumor necrosis factor-α and tumor necrosis factor receptor 1/tumor necrosis factor receptor 2 system in mononuclear cells recovered from peritoneal fluid of women with endometriosis at different stages. Reprod Sci.

